# Correction to “miR‐199a/214 cluster enhances prostate cancer sensitiveness to nimotuzumab via targeting TBL1XR1”

**DOI:** 10.1002/kjm2.12897

**Published:** 2024-09-19

Hu S , Zhou Q , Lu Q , Guo X , Wang Y , Duan Y‐X . miR‐199a/214 cluster enhances prostate cancer sensitiveness to nimotuzumab via targeting TBL1XR1. Kaohsiung J Med Sci 2023;39(12):1178–89. 10.1002/kjm2.12758 37772770 PMC11895912

We should have included the funding information stated below.

This work was supported by the Natural Science Funding‐Kewei Joint Project, Grant/Award Number: 2021JJ70015.

We apologize for this error.

